# Plasma-Derived Cell-Free DNA as a Biomarker for Early Detection, Prognostication, and Personalized Treatment of Urothelial Carcinoma

**DOI:** 10.3390/jcm13072057

**Published:** 2024-04-02

**Authors:** Sophia Bhalla, Rachel Passarelli, Antara Biswas, Subhajyoti De, Saum Ghodoussipour

**Affiliations:** 1 Division of Urology, Section of Urologic Oncology, Rutgers Cancer Institute of New Jersey, Rutgers Robert Wood Johnson University Hospital, 195 Albany St., New Brunswick, NJ 08901, USA; sjb343@rwjms.rutgers.edu (S.B.); rp1169@rwjms.rutgers.edu (R.P.); 2 Center for Systems and Computational Biology, Rutgers Cancer Institute of New Jersey, Rutgers University, 195 Albany St., New Brunswick, NJ 08901, USA; ab2063@cinj.rutgers.edu (A.B.); sd948@cinj.rutgers.edu (S.D.)

**Keywords:** biomarker, bladder cancer, cell-free DNA, circulating tumor DNA, urothelial carcinoma

## Abstract

Bladder cancer (BC) is one of the most common malignancies in the United States, with over 80,000 new cases and 16,000 deaths each year. Urothelial carcinoma (UC) is the most common histology and accounts for 90% of cases. BC management is complicated by recurrence rates of over 50% in both muscle-invasive and non-muscle-invasive bladder cancer. As such, the American Urological Association (AUA) recommends that patients undergo close surveillance during and after treatment. This surveillance is in the form of cystoscopy or imaging tests, which can be invasive and costly tests. Considering this, there have been recent pushes to find complements to bladder cancer surveillance. Cell-free DNA (CfDNA), or DNA released from dying cells, and circulating tumor DNA (ctDNA), or mutated DNA released from tumor cells, can be analyzed to detect and characterize the molecular characteristics of tumors. Research has shown promising results for ctDNA use in the BC care realm. A PubMed literature review was performed finding studies discussing cfDNA and ctDNA in BC detection, prognostication, and monitoring for recurrence. Keywords used included bladder cancer, cell-free DNA, circulating tumor DNA, urothelial carcinoma, and liquid biopsy. Studies show that ctDNA can serve as prognostic indicators of both early- and late-stage BC, aid in risk stratification prior to major surgery, assist in detection of disease progression and metastatic relapse, and can assess patients who may respond to immunotherapy. The benefit of ctDNA is not confined to BC, as studies have also suggested its promise as a biomarker for neoadjuvant chemotherapy in upper-tract UC. However, there are some limitations to ctDNA that require improvements in ctDNA-specific detection methods and BC-specific mutations before widespread utilization can be achieved. Further prospective, randomized trials are needed to elucidate the true potential ctDNA has in advancements in BC care.

## 1. Introduction

In 2023, it was estimated that more than 82,000 individuals would be diagnosed and over 16,000 would die from bladder cancer (BC) in the United States [1]. Although the overall 5-year survival rate is 77%, the 5-year survival rate for patients with metastatic disease is only 5% [2]. Tobacco smoke is the primary risk factor, contributing to the development of up to 50% of all bladder tumors. Other risk factors include chronic urinary tract infections, occupational exposure to carcinogens (e.g., aromatic amines and polycyclic aromatic hydrocarbons), chronic exposure to arsenic, pelvic radiation, and genetic predispositions [2,3,4,5,6]. Approximately 90% of BC cases are due to urothelial carcinoma (UC), which can affect the urothelial cells that line the entire urogenital tract, including the urethra, ureters, and renal pelvis [2].

Non-muscle-invasive bladder cancer (NMIBC) and muscle-invasive bladder cancer (MIBC) make up 70% and 30% of bladder cancer cases, respectively [7]. NMIBC typically has a favorable outcome after transurethral resection of bladder tumors (TURBT), with a 70–85% 10-year overall survival rate [8,9]. However, recurrence rates as high as 60–70% and progression rates of 10–20% necessitate intensive surveillance [8,9]. Up to 40–50% of high-grade NMIBC cases will progress to MIBC [10,11]. MIBC is treated with neoadjuvant chemotherapy followed by radical cystectomy and pelvic lymph node dissection, or concurrent chemotherapy and radiation. Despite these treatment efforts, 50% of MIBC patients will develop local or distant metastasis within two years of cystectomy [11,12].

The high risk of recurrence in both NMIBC and MIBC necessitates ongoing surveillance. Current guidelines suggest that patients with either NMIBC or MIBC who maintain intact bladders undergo lifelong surveillance with periodic cystoscopy [13,14]. This procedure is invasive and costly, posing a significant burden to the patient and to the healthcare system, with an estimated cost of over 5 billion dollars per year [15,16]. Patients without intact bladders are at risk for local and distant recurrence. It is recommended that these patients undergo chest, abdominal, and pelvic imaging via CT or MRI at 6–12-month intervals for several years, and then annually thereafter [13,17,18]. The high detection limit for these imaging techniques can result in a lag time between identification of disease recurrence and initiation of further therapy [19]. Adjuvant immunotherapy was recently approved to be administered post-cystectomy; however, there are no clear guidelines for this practice [20]. Clinicians’ current inability to detect early presentations and recurrence of BC is a major limitation in disease management. The development of an accurate, noninvasive, and affordable assay for BC detection would improve clinical management and overall survival (OS) in both NMIBC and MIBC. Liquid biopsy—sampling of non-solid tissue for tumor DNA—is a promising method to achieve this aim.

Precision oncology, the use of biologic tumor characteristics to elucidate molecularly targetable alterations, has drastically changed the landscape of cancer treatment over the past 30 years. While nonspecific cytotoxic agents remain the standard of care for most cancers, advancements in precision oncology have laid the foundation for targeted therapies currently approved for bladder cancer, including erdafitinib and enfortumab vedotin, and our ability to utilize ctDNA levels to track tumor burden in real time [21,22,23].

cfDNA is fragmented, extracellular DNA released from dying cells found in several bodily fluids, including serum and urine [24]. ctDNA is the portion of cfDNA that contains mutated DNA released from dying tumor cells. Over the past decade, ctDNA has gained attention for its potential to evaluate tumor burden in real time [25,26]. Its noninvasive method of collection allows for frequent sampling, which could lead to potential advancements in early cancer detection, assessment and monitorization of treatment efficacy, and detection of minimal residual disease. Circulating biomarkers, such as ctDNA, hold promise to address several unmet needs in BC management, including mechanisms for early detection and clinical staging, prognostication, and personalized treatment. Studies have shown that ctDNA is detectable in the plasma and urine of BC patients and that higher levels are associated with disease progression and metastasis [27,28,29]. ctDNA has also been studied in the treatment of upper-tract UC [30]. Successful use of liquid biopsy analysis in the treatment of BC, the most common and best-studied UC, could lead to improved management of UC throughout the genitourinary tract, including urethral and upper-tract disease. In this review, we aim to summarize findings from previous studies regarding cfDNA and ctDNA’s utility in BC management and to highlight ongoing clinical trials born from this data. UC of the bladder is the most common and best studied and, therefore, will be the focus of this review.

## 2. Methods

A narrative literature search was conducted on PubMed to identify studies assessing the roles of cfDNA and ctDNA in BC detection, prognostication, and monitoring for recurrence. Keywords used included bladder cancer, cell-free DNA, circulating tumor DNA, urothelial carcinoma, and liquid biopsy. We included original studies published from 2013 to 2024. We excluded non-English literature as well as case reports, reviews, and abstracts. A second search was conducted on clinicaltrials.gov for active trials involving cfDNA or ctDNA and UC management. We manually screened relevant studies. Those with cfDNA or ctDNA analysis in primary or secondary outcomes were included.

## 3. Results

We identified 38 publications that met our initial search criteria. After reviewing the full texts, 5 were included, and 15 were added based on expert opinion for a total of 20 studies. Of these, 18 commented on the ability of ctDNA to detect the presence of disease or recurrence and 12 on the prognostic capability of cfDNA.

The initial clinicaltrials.gov search yielded 37 studies. After screening, 15 studies were included, of which there were 3 randomized controlled trials, 3 single-arm clinical trials, 8 observational prospective cohort studies, and 1 cross-sectional case study.

## 4. Discussion

### 4.1. ctDNA Can Detect BC and Serve as a Prognostic Indicator in Early Disease Stages

Circulating tumor DNA has been studied as a potential detection method for bladder cancer (BC). Until recently, conclusions regarding the reliability of results of early studies have been variable. More recently, however, data have become more consistent, supporting the use of ctDNA as a diagnostic tool for bladder cancer detection. A 2019 meta-analysis evaluated 11 studies involving both early- and late-stage bladder cancer. Pooled sensitivity and specificity, respectively, were 0.69 (95%CI: 0.67, 0.71) and 0.72 (95%CI: 0.70, 0.74), and the area under summary receiver operating characteristic curve was 0.80 (95%CI: 0.77, 0.83). Although from a small sample size of 11 studies, these results are promising in suggesting the ctDNA assay as having high diagnostic value for BC detection [31].

Recent studies endorse the use of plasma ctDNA levels for early disease detection and as a prognostic indicator of disease-free survival (DFS) in cases of NMIBC and MIBC. Zhang et al. was the first group to study targeted sequencing of ctDNA in NMIBC. The 2021 study included 82 patients with stage Ta or T1 disease who underwent TURBT followed by adjuvant intravesical BCG immunotherapy. Somatic variations in plasma ctDNA were detected in 65% of patients. The molecular tumor burden index (mTBI), a reflection of the ctDNA fraction found in cfDNA, was positively correlated with tumor size and stage and was an independent predictor of recurrence. Higher mTBI levels were also associated with worse DFS. These authors revealed that ctDNA can be detected in patients with Ta NMIBC, suggesting that ctDNA can be used to detect bladder cancer in its earliest stages, possibly even before symptoms arise. The study also highlighted ctDNA’s ability to predict NMIBC aggressiveness and likelihood of recurrence in patients who underwent TURBT followed by BCG immunotherapy [32].

In addition to mTBI, future utilization of ctDNA as an indicator of early disease may involve specific hotspot mutation analysis. A 2020 study conducted by Hayashi et al. performed droplet digital PCR (ddPCR) analysis of hotspot gene mutations (TERT promoter and FGFR3) on urinary cfDNA from a cohort of 74 patients with UC. The sensitivity of urinary cfDNA diagnosis was 68.9%, and the specificity was 100%. Patients with a high TERT C228T allele frequency had significantly increased rates of recurrence after treatment [33]. Thus, ctDNA holds promise to add to the current arsenal for early detection and prognostication of BC.

### 4.2. ctDNA Can Risk-Stratify Patients before Extirpative Surgery

Emerging evidence suggests that cisplatin-based neoadjuvant chemotherapy (NAC) can lead to pathologic downstaging and improved survival in patients with aggressive urothelial carcinoma (UC) [34,35]. However, difficulties in obtaining accurate clinical staging before extirpative surgery cause missed opportunities for systemic therapy. This is especially important for patients who develop renal insufficiency with disease progression and become ineligible to receive NAC. Predictive biomarkers for invasive UC, such as ctDNA, could limit clinical downstaging, leading to increased DFS and OS.

A 2023 study published by Huelster et al. provided strong evidence that plasma ctDNA is highly prognostic for advanced-stage disease at the time of extirpative surgery. The prospective observational study was the first to investigate ctDNA’s ability to distinguish between patients with muscle-invasive (MI) and non-organ-confined (NOC) upper-tract urothelial carcinoma (UTUC) from those with non-muscle-invasive (NMI) UTUC in paired serum and tumor samples. The study included 30 patients diagnosed with resectable, clinically localized, high-risk UTUC scheduled for radical nephroureterectomy (RNU) or ureterectomy. Serum was collected 1–2 h prior to surgery. The presence of presurgical ctDNA was defined by detection of at least 2 plasma molecular alterations (MA) from a 152 gene panel that covers 81% of the commonly altered genes in UTUC. At least one MA was found in 70% of perioperative plasma samples and 97% of tumor samples, and 52% of serum ctDNA variants were found in matching tumor samples. ctDNA-positive status was strongly predictive of MI/NOC UTUC at the time of surgery, with a sensitivity of 71% and a specificity of 94%. Patients with presurgical ctDNA had worse outcomes. Of the 11 ctDNA-positive patients, 5 experienced progression, 3 died from UTUC, and 1 died from an unknown cause, whereas only 1 ctDNA-negative patient experienced disease progression and death [36].

This study highlights ctDNA’s potential to accurately stage UC. Most serum mutations were found in both serum and tumor samples, highlighting ctDNA’s ability to assess tumor burden in real time. While neoadjuvant therapies are approved, there are no clear guidelines for this practice. A standardized method to determine ctDNA-positive or -negative status and, therefore, which patients could benefit from NAC, would reduce missed opportunities for systemic therapy and improve DFS and OS in aggressive UC.

While NAC can lead to pathologic downstaging and improved OS, the treatment is often intense. Optimally, only patients who would benefit would receive treatment. Early studies have indicated that cfDNA methylation can be used to generate classifiers of NAC response in BC patients. In a 2023 study, the pathologic response to NAC was correctly predicted in 79% of blood samples using a model combining the methylation-based response score (mR-score) and circulating bladder DNA fraction [37]. These studies highlight cfDNA analysis’ promise to determine which patients would benefit from NAC in the treatment of BC.

### 4.3. ctDNA Analysis Can Detect Metastatic Relapse and Predict Disease Progression of MIBC

In addition to detection of organ-confined BC, plasma-derived ctDNA has been shown to predict disease progression and provide early detection of metastatic disease in patients with MIBC. In a longitudinal study published in 2019, Christensen et al. enrolled 68 patients with organ-confined MIBC who received NAC before cystectomy. Plasma ctDNA was assessed via ultra-deep sequencing at four time points: at diagnosis, during chemotherapy, before cystectomy, and during surveillance. Patient samples were determined as ctDNA-positive or ctDNA-negative based on a previously validated method. Positive or negative ctDNA status was strongly associated with patient outcomes. Patients with ctDNA-positive samples before chemotherapy, before cystectomy, and during surveillance had lower DFS and OS compared to ctDNA-negative patients at the same time points. Recurrence occurred in 46% of patients who were ctDNA-positive before chemotherapy, compared to only 2% in patients who were ctDNA-negative at this time point. All patients who were ctDNA-positive before cystectomy had either residual tumor (≥T1) or lymph node metastasis at cystectomy, and 100% of patients who were pT0 at cystectomy were ctDNA-negative. ctDNA status at any time after cystectomy was the strongest predictive factor for metastatic recurrence. None of the 47 patients who were ctDNA-negative during surveillance experienced metastatic relapse. ctDNA correctly identified 100% of patients who relapsed during surveillance, with a median lead time of 96 days over imaging, the current standard of care [38]. These results highlight ctDNA analysis’ potential to detect aggressive cases during early stages of MIBC treatment, as positive ctDNA status at all time points was associated with worse DFS and OS.

Christensen et al.’s study results indicated ctDNA’s immense potential to personalize the treatment of BC. For example, patients who are ctDNA-positive prior to cystectomy may undergo additional treatment prior to surgery or consider alternative therapies to improve oncologic outcomes. Post-cystectomy, ctDNA-negative patients, who have a lower probability of metastatic relapse, could be spared the side effects of adjuvant therapies. Similarly, physicians could provide ctDNA-positive patients, who are at high risk of metastatic recurrence, adjuvant therapies post-cystectomy. ctDNA’s ability to detect disease earlier than conventional imaging techniques provides an avenue for an improved, less invasive, and less expensive form of BC surveillance in patients at all time points post-cystectomy. When combined with Zhang et al. and others’ findings, there is a strong case for ctDNA analysis’ ability to serve as a noninvasive, effective, and less costly mechanism for BC surveillance in patients with a history of both NMIBC and MIBC.

### 4.4. ctDNA Status Can Identify Patients Who May Respond to Immunotherapy

Due to the high rate of metastatic relapse post-cystectomy, adjuvant immunotherapies—agents designed to elicit or amplify immune responses to treat disease—are sometimes recommended to prolong DFS and OS. There are currently no clear guidelines to dictate which patients should receive such therapy [39]. Two monoclonal antibodies, nivolumab and atezolizumab, have been studied as adjuvant therapies for MIBC.

Adjuvant nivolumab treatment was evaluated in the Checkmate 274 study, a phase 3 multicenter, double-blind, randomized controlled trial of 709 patients that evaluated adjuvant nivolumab versus placebo in patients with muscle-invasive urothelial carcinoma who underwent cystectomy. DFS was significantly longer in patients receiving nivolumab compared to placebo [40]. This led to FDA approval for adjuvant immunotherapy for patients with MIBC with a high risk of recurrence, defined as those with residual muscle-invasive disease after chemotherapy, extravesical tumor extension, or node-positive disease.

IMvigor010, a phase 3, multicenter, randomized trial that enrolled over 800 patients over three years, evaluated adjuvant atezolizumab versus observation in patients with muscle-invasive urothelial carcinoma. The trial did not meet its primary endpoint, as neither DFS nor OS was improved with adjuvant atezolizumab compared to observation [41]. Subsequent analysis of the data, however, revealed that patients who were ctDNA-positive post-cystectomy had improved DFS and OS with atezolizumab compared to observation only [42]. This finding inspired the ongoing IMvigor011 trial to evaluate adjuvant atezolizumab in ctDNA-positive patients post-cystectomy [43].

Although nivolumab has been approved by the FDA, there are no clear guidelines that outline which patients should receive it. Data from IMvigor010 suggests ctDNA-positive or -negative status could be used to identify patients who would benefit most from adjuvant immunotherapy. This highlights another use for plasma-derived ctDNA in BC treatment. Stratification of patients based on ctDNA status holds the potential to spare patients with a lower risk of recurrence from continued treatment and immunotherapy side effects and provide additional therapy only to those with a high risk of metastatic recurrence to improve DFS and OS.

The correlation between the response to Durvulamab in urothelial cancer patients and positive ctDNA assays has also been assessed. In a retrospective study, Raja et al. evaluated 29 patients with urothelial cancer, and two cohorts of patients with non-small-cell lung cancer to correlate ctDNA responses to Durvulamab treatment. Overall, they identified that changes in ctDNA frequency preceded radiographic regression and were correlated with longer progression-free and overall survival [44].

## 5. Challenges with cfDNA Use

The ability to distinguish ctDNA from cfDNA released from healthy cells is key to ctDNA’s use as a biomarker in oncology. Capturing non-tumor DNA is a major limitation in the use of liquid biopsy in BC management. This task is made challenging by ctDNA’s short fragments, small volume compared to cfDNA, and the fact that benign lesions can produce ctDNA identical to malignant lesions [24,25,45]. Additionally, multiple cancer types can harbor the same mutations, making it difficult to distinguish the source of plasma-derived ctDNA [24]. In bladder cancer specifically, the high heterogeneity of tumors makes defining a specific set of mutations for detection a challenge [10]. Improvements in ctDNA-specific detection methods and understanding of BC-specific mutations will help the field effectively utilize ctDNA technology for the detection and treatment of BC.

## 6. Conclusions and Future Directions

BC is one of the most common and lethal malignancies in the United States. Care for patients is made difficult due to limitations in detection mechanisms, risk stratification, and ambiguous guidelines for adjuvant therapy. Plasma-derived ctDNA has emerged as a promising biomarker to provide a quick, repeatable, and minimally invasive assay to evaluate patients for BC diagnosis, disease progression, and metastatic relapse. Preliminary studies support ctDNA’s ability to detect BC at all disease stages, identify aggressive cases, and predict which patients would benefit from adjuvant therapies post-cystectomy. Next steps for the field include validation of these results through prospective, randomized clinical trials to further showcase ctDNA’s promise as a transformative advance for cancer medicine (Table 1).

## Figures and Tables

**Table 1:** Active clinical trials using ctDNA analysis in urothelial carcinoma.

Trial	Sample Size	Patient Population	Intervention	Relevant Objectives
Randomized controlled trials				
ANTICIPATE (NCT04813107)	79	Patients with MIBC who refused or were ineligible for cisplatin-based NAC and will undergo radical cystectomy.	Active Comparator: APL-1202 and tislelizumab Placebo Comparator: Tiselizumab alone	To assess tumor mutational burdens in pre- and post-treatment cfDNA, ctDNA, and tumor tissue.
IMvigor011 (NCT04660344)	520	Patients with high-risk MIBC who are ctDNA-positive following cystectomy.	Atezolizumab	DFS within 24 weeks of cystectomy.
MODERN (NCT05987241)	1190	Bladder cancer patients post-cystectomy.	Cohort A: ctDNA-positive Arm 1: Nivolumab Arm 2: Nivolumab + retalimab Cohort B: ctDNA-negative Arm 3: Nivolumab Arm 4: ctDNA surveillance	1. To compare ctDNA clearance in Cohort A 2. To compare OS in Cohort A 3. To compare DFS in Cohort B
Single-arm clinical trials				
CLONEVO (NCT03837821)	20	MIBC patients who refused or are ineligible for cisplatin-based chemotherapy.	Abemaciclib followed by radical cystectomy	To measure changes in cell-cycle dynamics pre- and post-Abemaciclib in tumor samples and ctDNA from blood.
RESPONDER (NCT03263039)	80	Patients with urothelial carcinoma that has progressed after platinum-containing chemotherapy.	Pembrolizumab	To collect longitudinal blood samples for isolation of immune cells, cytokine/chemoattractants, and ctDNA.
TOMBOLA (NCT04138628)	282	ctDNA-positive patients who have undergone radical cystectomy due to MIBC without metastases following NAC.	Atezolizumab	To evaluate atezolizumab’s ability to achieve complete response after treatment.
Observational prospective cohort studies				
Assessment of the Concordance of Genomic Alterations Between Urine and Tissue in High-Risk NMIBC Patients (NCT04412070)	40	Patients with NMIBC before BCG treatment and MIBC before radical cystectomy.	N/A	To assess the agreement rate between urine cell-free DNA and tumor tissue mutation profile.
Circulating Tumor DNA and Urine Tumor DNA Detection of Minimal Residual Disease in Locally Advanced Upper-Tract Urothelial Carcinoma with Radical Nephroureterectomy: A Cohort Study (NCT05595408)	103	Patients with muscle-invasive upper-tract urothelial carcinoma after radical nephroureterectomy receiving adjuvant chemotherapy or adjuvant immunotherapy.	N/A	To assess the predictive value of ctDNA and utDNA.
Circulating Tumor DNA Exposure in Peripheral Blood (NCT03517332)	10,000	Patients with a diagnosed malignancy (including, but not limited to, colorectal, pancreatic, gastric, hepatocellular carcinoma, non-small-cell lung cancer, bladder, and melanoma) and subjects that have never been diagnosed with malignancy.	N/A	To test the feasibility of the detection of tumor DNA in peripheral blood using a novel process for the detection of ctDNA.
MARIA (NCT05219734)	400	Patients with solid tumors, with emphasis on early-stage non-metastatic colorectal, non-small-cell lung cancer, breast, ovarian, and bladder cancers.	Personalized molecular testing to detect the presence of ctDNA and MRD	To evaluate DFS and OS.
Clinical Performance Evaluation of the C2i Test (NCT05860543)	125	Patients with MIBC who will undergo radical cystectomy.	C2i-WGS-MRD Test (personalized molecular ctDNA test)	To assess the specificity of the C2i-WGS-MRD Test in predicting 2-year recurrence-free survival.
ORACLE (NCT05059444)	1000	Patients with invasive bladder, ureteral, or renal pelvis carcinoma, NSCLC, or breast cancer with residual invasive disease following neoadjuvant chemotherapy.	Guardant Reveal (MRD panel for use in recurrence detection)	To evaluate distant recurrence-free interval.
ctDNA in Subjects with Muscle-Invasive Bladder Cancer Treated with Trimodality Therapy (NCT5630131)	20	Patients with MIBC to be treated with surgery, radiation, and chemotherapy.	N/A	To evaluate the feasibility of measuring ctDNA in serum and urine in patients with MIBC treated with trimodality therapy.
Preventing Viral Pandemic-Associated Risk of Cancer Death Using Less Invasive Diagnostic Tests—Liquid Biopsies (NCT04566614)	294	Patients with suspected malignancy (biliary tract, bladder, pancreatic, gastrointestinal stromal, lung, and colorectal) for whom invasive biopsy for definitive histological diagnosis is challenging due to COVID-19-related resource limitations, infection control, or technical feasibility.	N/A	To investigate the feasibility of using ctDNA to support cancer diagnosis and risk stratification.
Cross-sectional case study				
ctDNA-FGFR Status as a Predictive Biomarker for FGFR-Targeted Therapy (NCT06129084)	210	Metastatic BC patients who have archival tissue sent for FGFR testing.	N/A	To evaluate the diagnostic value of ctDNA testing for FGFR against standard tissue testing.

## Data Availability

All data are publicly available.

## References

[1] American Cancer Society Key Statistics for Bladder Cancer. Updated 13 January 2023. https://www.cancer.org/cancer/types/bladder-cancer/about/key-statistics.html.

[2] Saginala K., Barsouk A., Aluru J.S., Rawla P., Padala S.A., Barsouk A. (2020). Epidemiology of Bladder Cancer. Med. Sci..

[3] Rothman N., Garcia-Closas M., Chatterjee N., Malats N., Wu X.F., Figueroa J.D., Real F.X., Van den Berg D., Matullo G., Baris D. (2010). A multi-stage genome-wide association study of bladder cancer identifies multiple susceptibility loci. Nat. Genet..

[4] Freedman N.D., Silverman D.T., Hollenbeck A.R., Schatzkin A., Abnet C.C. (2011). Association between smoking and risk of bladder cancer among men and women. JAMA.

[5] Brandau S., Böhle A. (2001). Bladder cancer. I. Molecular and genetic basis of carcinogenesis. Eur. Urol..

[6] Abern M.R., Dude A.M., Tsivian M., Coogan C.L. (2013). The characteristics of bladder cancer after radiotherapy for prostate cancer. Urol. Oncol..

[7] Kaufman D.S., Shipley W.U., Feldman A.S. (2009). Bladder cancer. Lancet.

[8] Slovacek H., Zhuo J., Taylor J.M. (2021). Approaches to Non-Muscle-Invasive Bladder Cancer. Curr. Oncol. Rep..

[9] Teoh J.Y., Kamat A.M., Black P.C., Grivas P., Shariat S.F., Babjuk M. (2022). Recurrence mechanisms of non-muscle-invasive bladder cancer—A clinical perspective. Nat. Rev. Urol..

[10] Carrasco R., Ingelmo-Torres M., Gómez A., Trullas R., Roldán F.L., Ajami T., Moreno D., Rodríguez-Carunchio L., Alcaraz A., Izquierdo L. (2022). Cell-Free DNA as a Prognostic Biomarker for Monitoring Muscle-Invasive Bladder Cancer. Int. J. Mol. Sci..

[11] Sylvester R.J., van der Meijden A.P., Oosterlinck W., Witjes J.A., Bouffioux C., Denis L., Newling D.W., Kurth K. (2006). Predicting recurrence and progression in individual patients with stage Ta T1 bladder cancer using EORTC risk tables: A combined analysis of 2596 patients from seven EORTC trials. Eur. Urol..

[12] del Pozo Jiménez G., Amo F.H., Arija J.A., Fernández E.R., Ríos D.S., García E.L., Chomón G.B., Gil M.C., Rodríguez J.C., Fernández C.H. (2020). Effect of adjuvant chemotherapy in locally advanced urothelial carcinoma of the bladder treated with cystectomy. Efecto de la quimioterapia adyuvante en el carcinoma urotelial de vejiga localmente avanzado tratado con cistectomía. Actas Urol. Esp. (Engl. Ed.).

[13] Chang S.S., Bochner B.H., Chou R., Dreicer R., Kamat A.M., Lerner S.P., Lotan Y., Meeks J.J., Michalski J.M., Morgan T.M. (2017). Treatment of Non-Metastatic Muscle-Invasive Bladder Cancer: AUA/ASCO/ASTRO/SUO Guideline. J. Urol..

[14] Power N.E., Izawa J. (2016). Comparison of Guidelines on Non-Muscle Invasive Bladder Cancer (EAU, CUA, AUA, NCCN, NICE). Bladder Cancer..

[15] Svatek R.S., Hollenbeck B.K., Holmäng S., Lee R., Kim S.P., Stenzl A., Lotan Y. (2014). The economics of bladder cancer: Costs and considerations of caring for this disease. Eur. Urol..

[16] Yeung C., Dinh T., Lee J. (2014). The health economics of bladder cancer: An updated review of the published literature. Pharmacoeconomics.

[17] Cagiannos I., Morash C. (2009). Surveillance strategies after definitive therapy of invasive bladder cancer. Can. Urol. Assoc. J..

[18] Wong V.K., Ganeshan D., Jensen C.T., Devine C.E. (2021). Imaging and Management of Bladder Cancer. Cancers.

[19] Erdi Y.E. (2012). Limits of Tumor Detectability in Nuclear Medicine and PET. Mol. Imaging Radionucl. Ther..

[20] Kim I.H., Lee H.J. (2020). Perioperative immunotherapy for muscle-invasive bladder cancer. Transl. Androl. Urol..

[21] Doroshow D.B., Doroshow J.H. (2020). Genomics and the History of Precision Oncology. Surg. Oncol. Clin. N. Am..

[22] Loriot Y., Necchi A., Park S.H., Garcia-Donas J., Huddart R., Burgess E., Fleming M., Rezazadeh A., Mellado B., Varlamov S. (2019). Erdafitinib in Locally Advanced or Metastatic Urothelial Carcinoma. N. Engl. J. Med..

[23] Powles T., Rosenberg J.E., Sonpavde G.P., Loriot Y., Durán I., Lee J.-L., Matsubara N., Vulsteke C., Castellano D., Wu C. (2021). Enfortumab Vedotin in Previously Treated Advanced Urothelial Carcinoma. N. Engl. J. Med..

[24] Song P., Wu L.R., Yan Y.H., Zhang J.X., Chu T., Kwong L.N., Patel A.A., Zhang D.Y. (2022). Limitations and opportunities of technologies for the analysis of cell-free DNA in cancer diagnostics. Nat. Biomed. Eng..

[25] Lin C., Liu X., Zheng B., Ke R., Tzeng C.M. (2021). Liquid Biopsy, ctDNA Diagnosis through NGS. Life.

[26] Pessoa L.S., Heringer M., Ferrer V.P. (2020). ctDNA as a cancer biomarker: A broad overview. Crit. Rev. Oncol. Hematol..

[27] Birkenkamp-Demtröder K., Nordentoft I., Christensen E., Høyer S., Reinert T., Vang S., Borre M., Agerbaek M., Jensen J.B., Ørntoft T.F. (2016). Genomic Alterations in Liquid Biopsies from Patients with Bladder Cancer. Eur. Urol..

[28] Birkenkamp-Demtröder K., Christensen E., Nordentoft I., Knudsen M., Taber A., Høyer S., Lamy P., Agerbæk M., Jensen J.B., Dyrskjøt L. (2018). Monitoring Treatment Response and Metastatic Relapse in Advanced Bladder Cancer by Liquid Biopsy Analysis. Eur. Urol..

[29] Patel K.M., van der Vos K.E., Smith C.G., Mouliere F., Tsui D., Morris J., Chandrananda D., Marass F., Van Den Broek D., Neal D. (2017). Association Of Plasma And Urinary Mutant DNA With Clinical Outcomes In Muscle Invasive Bladder Cancer. Sci. Rep..

[30] Rose K.M., Huelster H.L., Meeks J.J., Faltas B.M., Sonpavde G.P., Lerner S.P., Ross J.S., Spiess P.E., Grass G.D., Jain R.K. (2023). Circulating and urinary tumour DNA in urothelial carcinoma—Upper tract, lower tract and metastatic disease. Nat. Rev. Urol..

[31] Cao Z., Peng L., He K., Faltas B.M., Sonpavde G.P., Lerner S.P., Ross J.S., Spiess P.E., Grass G.D., Jain R.K. (2019). Value of quantitative and qualitative analyses of serum and urine cell-free DNA as diagnostic tools for bladder cancer: A meta-analysis. Expert Rev. Anticancer Ther..

[32] Zhang J., Dai D., Tian J., Li L., Bai J., Xu Y., Wang Z., Tang A. (2021). Circulating Tumor DNA Analyses Predict Disease Recurrence in Non-Muscle-Invasive Bladder Cancer. Front. Oncol..

[33] Hayashi Y., Fujita K., Matsuzaki K., Eich M.L., Tomiyama E., Matsushita M., Koh Y., Nakano K., Wang C., Ishizuya Y. (2020). Clinical Significance of Hotspot Mutation Analysis of Urinary Cell-Free DNA in Urothelial Bladder Cancer. Front. Oncol..

[34] Coleman J.A., Yip W., Wong N.C., Sjoberg D.D., Bochner B.H., Dalbagni G., Donat S.M., Herr H.W., Cha E.K., Donahue T.F. (2023). Multicenter Phase II Clinical Trial of Gemcitabine and Cisplatin as Neoadjuvant Chemotherapy for Patients With High-Grade Upper Tract Urothelial Carcinoma. J. Clin. Oncol..

[35] Margulis V., Puligandla M., Trabulsi E.J., Plimack E.R., Kessler E.R., Matin S.F., Godoy G., Alva A., Hahn N.M., Carducci M.A. (2020). Phase II Trial of Neoadjuvant Systemic Chemotherapy Followed by Extirpative Surgery in Patients with High Grade Upper Tract Urothelial Carcinoma. J. Urol..

[36] Huelster H.L., Gould B., Schiftan E.A., Camperlengo L., Davaro F., Rose K.M., Soupir A.C., Jia S., Zheng T., Sexton W.J. (2023). Novel Use of Circulating Tumor DNA to Identify Muscle-invasive and Non-organ-confined Upper Tract Urothelial Carcinoma. Eur. Urol..

[37] Lu Y.T., Plets M., Morrison G., Cunha A.T., Cen S.Y., Rhie S.K., Siegmund K.D., Daneshmand S., Quinn D.I., Meeks J.J. (2023). Cell-free DNA Methylation as a Predictive Biomarker of Response to Neoadjuvant Chemotherapy for Patients with Muscle-invasive Bladder Cancer in SWOG S1314. Eur. Urol. Oncol..

[38] Christensen E., Birkenkamp-Demtröder K., Sethi H., Shchegrova S., Salari R., Nordentoft I., Wu H.-T., Knudsen M., Lamy P., Lindskrog S.V. (2019). Early Detection of Metastatic Relapse and Monitoring of Therapeutic Efficacy by Ultra-Deep Sequencing of Plasma Cell-Free DNA in Patients With Urothelial Bladder Carcinoma. J. Clin. Oncol..

[39] Alevizakos M., Bellmunt J. (2022). Adjuvant immunotherapy for muscle-invasive urothelial carcinoma of the bladder. Expert Rev. Anticancer Ther..

[40] Bajorin D.F., Witjes J.A., Gschwend J.E., Schenker M., Valderrama B.P., Tomita Y., Bamias A., Lebret T., Shariat S.F., Park S.H. (2021). Adjuvant Nivolumab versus Placebo in Muscle-Invasive Urothelial Carcinoma. N. Engl. J. Med..

[41] Bellmunt J., Hussain M., Gschwend J.E., Albers P., Oudard S., Castellano D., Daneshmand S., Nishiyama H., Majchrowicz M., Degaonkar V. (2021). Adjuvant atezolizumab versus observation in muscle-invasive urothelial carcinoma (IMvigor010): A multicentre, open-label, randomised, phase 3 trial. Lancet Oncol..

[42] Powles T., Assaf Z.J., Davarpanah N., Banchereau R., Szabados B.E., Yuen K.C., Grivas P., Hussain M., Oudard S., Gschwend J.E. (2021). ctDNA guiding adjuvant immunotherapy in urothelial carcinoma. Nature.

[43] Jackson-Spence F., Toms C., O’Mahony L.F., Choy J., Flanders L., Szabados B., Powles T. (2023). IMvigor011: A study of adjuvant atezolizumab in patients with high-risk MIBC who are ctDNA+ post-surgery. Future Oncol..

[44] Raja R., Kuziora M., Brohawn P.Z., Higgs B.W., Gupta A., Dennis P.A., Ranade K. (2018). Early Reduction in ctDNA Predicts Survival in Patients with Lung and Bladder Cancer Treated with Durvalumab. Clin. Cancer Res..

[45] Gilson P. (2020). Enrichment and Analysis of ctDNA. Recent Results Cancer Res..

